# Automatic Detection of the EEG Spike–Wave Patterns in Epilepsy: Evaluation of the Effects of Transcranial Current Stimulation Therapy

**DOI:** 10.3390/ijms25169122

**Published:** 2024-08-22

**Authors:** Elzbieta Olejarczyk, Aleksander Sobieszek, Giovanni Assenza

**Affiliations:** 1 Nalecz Institute of Biocybernetics and Biomedical Engineering, Polish Academy of Sciences, 02-109 Warsaw, Poland; 2 Independent Researcher, 02-797 Warsaw, Poland; 3 Operative Research Unit of Neurology, Fondazione Policlinico Universitario Campus Bio-Medico, Via Alvaro del Portillo, 200, 00128 Roma, Italy; 4 Research Unit of Neurology, Neurophysiology and Neurobiology, Department of Medicine and Surgery, Università Campus Bio-Medico di Roma, Via Alvaro del Portillo, 21, 00128 Roma, Italy

**Keywords:** epilepsy, electroencephalography (EEG), epileptiform activity, spike–wave (SW) patterns, morphological features of EEG patterns, cathodal transcranial direct current stimulation (ctDCS)

## Abstract

This study aims to develop a detection method based on morphological features of spike–wave (SW) patterns in the EEG of epilepsy patients and evaluate the effect of cathodal transcranial direct current stimulation (ctDCS) treatment. The proposed method is based on several simple features describing the shape of SW patterns and their synchronous occurrence on at least two EEG channels. High sensitivity, specificity and selectivity values were achieved for each patient and condition. ctDCS resulted in a significant reduction in the number of detected patterns, a decrease in spike duration and amplitude, and an increased spike mobility. The proposed method allows efficient identification of SW patterns regardless of brain condition, although the recruitment of patterns may be modified by ctDCS. This method can be useful in the clinical evaluation of ctDCS effects.

## 1. Introduction

Epilepsy is one of the most common neurological disorders worldwide. According to the World Health Organization, the number of people with epilepsy ranks fourth after strokes and Alzheimer’s and Parkinson’s diseases, and it is estimated to be around 50 million people. Epilepsy is characterized by a predisposition to generate seizures as a result of an abnormal excessive or synchronous neuronal activity in the brain [1,2]. This epileptic activity is diagnosed using electroencephalography (EEG). Spikes and spike–wave (SW) are EEG epileptiform wave patterns specifically associated with epilepsy. Spike is defined as a transient, clearly distinguished from background activity, with a pointed peak at a conventional time scale and duration from 20 to less than 70 ms [3]. An interictal activity manifested as spike–wave patterns in EEG indicates cortical hyperexcitability, which can lead to a seizure. Thus, its detection is helpful in the localization of seizure onset. A standard EEG recording of a person with epilepsy can show several SWs, and their identification is a challenging and time-consuming task for a neurologist. At the end of the 1970s, the development of modern computer systems and methods allowed to develop methods of automatic detection of SW patterns. For the first time, Gotman and Gloor [4] proposed an algorithm based on morphological features of spikes to detect epileptiform EEG patterns. They calculated the spike duration as well as the ratios of amplitudes and durations of the rising and falling slope of the spike, respectively. Moreover, the relative sharpness of both slopes, calculated as their second derivative, was included as an additional feature. All these features were normalized in relation to their corresponding values for background. Subsequently, Gotman et al. [5,6,7,8] made several extensions to this algorithm, allowing for the rejection of physiological artifacts like EMG, eyeblinks, alpha waves, and sleep-related patterns like vertex sharp transients and spindles. Further modifications to this method were described in the reviews of Wilson and Emerson [9] and Harner [10]. The application of new technologies, such as artificial neural networks [11,12] or wavelet transform [12,13], did not improve the accuracy of the spike detection. Liu et al. [12] proposed a system integrating several methods, including adaptive filtering, wavelet transform, artificial neural network and expert system. They also emphasized the importance of slow wave features in detecting SW patterns. Several observations of other authors are also noteworthy. Harner [10] highlighted the significance of EEG normalization due to considerable inter-individual signal variability and its dependence on brain state and EEG channel or source localization. Moreover, Frost [14] pointed out the need to consider a wide temporal and spatial context in EEG analysis to reduce the high false-positive rate of spikes as early as 1985. Interestingly, the SW patterns similar to that observed in epilepsy occur in the EEG of healthy individuals undergoing volatile anesthesia [15,16,17,18,19,20]. Olejarczyk et al. [21] proposed a method of automatic detection and analysis of spike/sharp wave–slow wave (SWSW) patterns based on the morphology of slow waves and their synchronization on at least two EEG channels. Then, the eighteen morphological features of patterns indicated by an expert were used as a reference set in the k-NN classification. Considering the features of slow waves rather than spikes and a wide spatial context expressed by synchronization of EEG activity on several channels was crucial for achieving high accuracy in this method. The following year, Liu et al. [22] modified their previous method [12] using the k-point nonlinear energy operator to detect potential spikes. Then, thirteen features, such as durations, amplitudes, slopes, and areas of potential spikes and slow waves, or their half-waves, and the sharpness of spikes, were used in the AdaBoost classification. Shibasaki et al. [23] extended the feature set by including the ratio of the slow wave amplitude to the average amplitude of background activity. Chang et al. [24] applied the features defined by Olejarczyk et al. [21] for detecting SW patterns in EEG recorded in patients with epilepsy. However, the procedure for potential pattern identification was changed. This algorithm determined the area between the signal and line drawn from a local maximum to a moving point near this maximum with a value less than a predetermined threshold. The support vector machine was used for pattern classification. The sensitivity, specificity and selectivity of this method were equal to 94.02, 93.50 and 93.51, respectively. However, the authors did not consider the condition for synchronization of patterns on at least two EEG channels.

In this study, we propose a simple method of automatic detection of SW patterns in epileptic EEG. The algorithm is a modification of our previous method used to detect similar patterns in volatile anesthesia. However, the identification of SW patterns in epilepsy appears to be simpler and allows for greater accuracy. We introduced a similarity index to evaluate the variability of morphological features of the SW patterns in individual patients’ EEG signals. Moreover, the impact of standardization and min–max normalization on the classification performance was tested.

The determination of the number of SW patterns and their features allowed for evaluating the effect of cathodal transcranial direct current stimulation (ctDCS). A decrease in the number of SW patterns was observed after stimulation. The spike duration and amplitude and the ratio between the amplitudes of rising and falling parts of the spike decreased, while spike mobility increased after ctDCS.

## 2. Results

### 2.1. The Impact of Choice of the Reference Electrode on SW Patterns Identification

It should be emphasized that the interpretation and identification of EEG patterns depend strongly on the choice of reference electrode. The base montage used during the EEG recording should always be applied to obtain a correct assessment.

**Figure 1:**
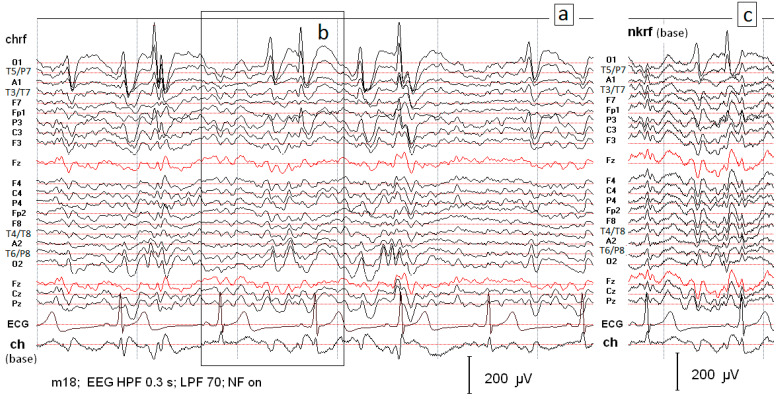
(**a**) Segment of the EEG record with characteristic spike–wave patterns received using the reference electrode located on the chin. The record (**c**) presents the same data as those illustrated in (**b**) but recorded using the reference electrode located at the neck.

**Figure 2:**
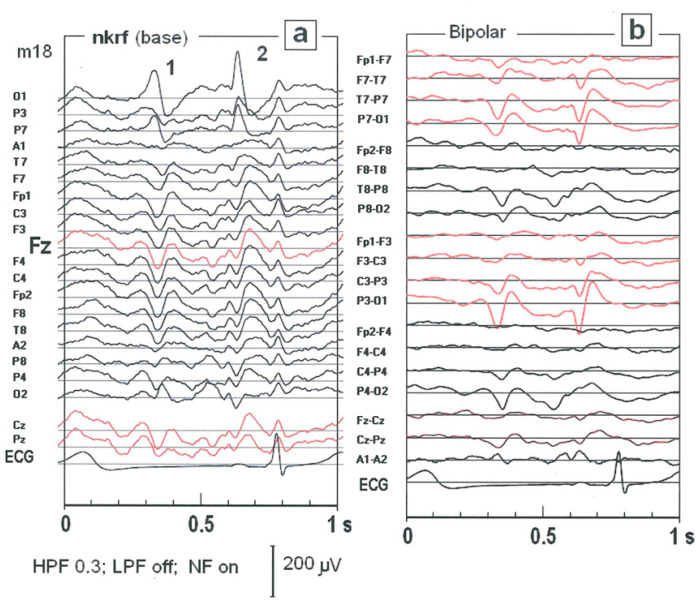
A one-second segment of the EEG record is presented in Figure 1c. Two montages are compared for the same EEG fragment: the basic montage with the reference electrode at the neck (**a**) and the bipolar montage (**b**).

An example of an EEG signal with characteristic SW patterns is shown in Figure 1. Two montages are compared for the EEG signal in P2: the montage with the reference electrode at the chin (Figure 1a) and the base montage with the reference electrode at the neck (Figure 1b). The algorithm was constructed for the SW patterns shown in Figure 2a. Although the spike amplitude is lower in the base montage, it guarantees more reliable signal evaluation because the important information is not lost. In Figure 1b, the ECG artifact can be observed at the beginning of the segment, which was not seen in other montages. Moreover, the potential distribution of the electric dipole field in the whole brain is well expressed. The negative pole is localized in the left occipital lobe at O1, corresponding to the maximal value of spike amplitude, while the positive pole is situated in the right frontotemporal part of the brain.

**Figure 3:**
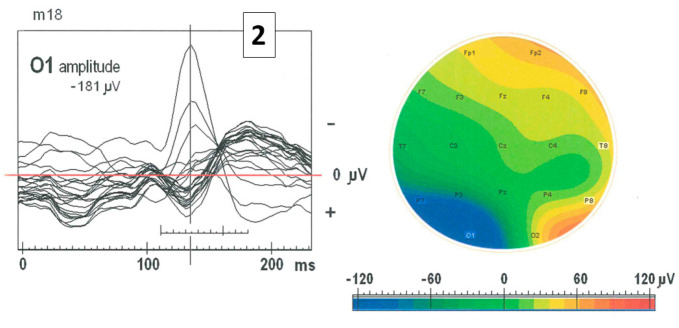
The plot presents the superimposition of SW patterns from all EEG channels at the position marked in Figure 2a with the number 2. The SW pattern at electrode O1 has the largest negative amplitude of −181 μV and lasts about 70 ms. The topographical map illustrates that the SW pattern with high negative amplitude at O1 is marked with blue color, while the highest positive amplitudes are marked with red color in the right hemisphere at Fp2, F8, P8, O2.

In clinical practice, standard bipolar montage is often used. However, the shape of SW patterns is completely different in the bipolar montage compared to the base one (see Figure 2). In Figure 3, the superimposition of SW patterns from all EEG channels and the topographical map of amplitude are shown. The SW pattern at the electrode O1 has the greatest amplitude (−181 μV) and lasts about 70 ms.

**Figure 4:**
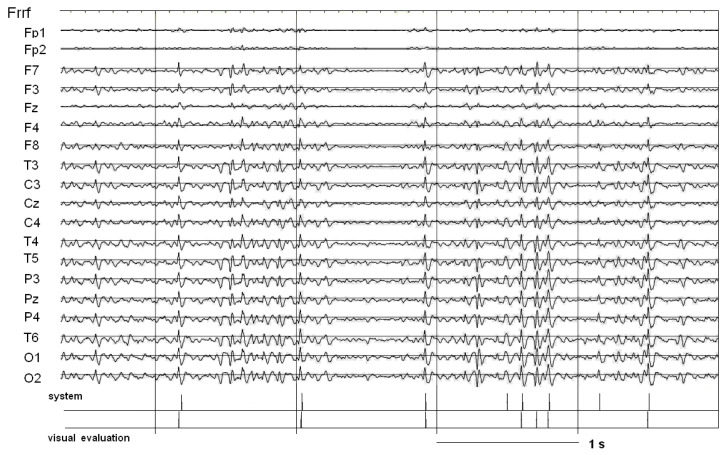
About five-second EEG segment with SW patterns indicated by expert and/or identified by the system in patient P1 in condition PRE STIM.

A wider variability of the SW patterns was observed in patient P1 than in P2 (see Figure 4). The high performance of the system is manifested in a high agreement between the occurrences of both patterns, those indicated by experts and the patterns identified by the system.

### 2.2. The Performance of SW Patterns Classification

The proposed method of the identification of SW patterns was validated by means of sensitivity, specificity and selectivity, which are defined in Appendix A. The values of these measures obtained for two patients (P1 and P2) are shown in Table 1. For patient P1, the EEG recording was evaluated in two conditions: before and after ctDCS.

### 2.3. Impact of Standardization on Classification Performance

The combination of standardization and min–max normalization allowed for obtaining a high classification performance in patient P2. The sensitivity (S1), selectivity (S2) and specificity (S3) were equal to 0.93, 0.91 and 1.00, respectively (marked with gray in Table 1). No pattern was found applying min–max normalization without standardization, while just the same approach gave the best results in patient P1 in both conditions (before ctDCS: S1 = 0.91, S2 = 0.90 and S3 = 1.00; after ctDCS: S1 = 0.89, S2 = 0.90 and S3 = 1.00) (marked with gray in Table 1). The S1 significantly decreased and S2 increased when the standardization was applied in patient P1 independently on the condition (before ctDCS: S1 = 0.70, S2 = 0.94 and S3 = 1.00; after ctDCS: S1 = 0.72, S2 = 0.95 and S3 = 1.00).

### 2.4. SW Patterns Similarity Testing

The distribution of the similarity index was much wider in P1 than in P2 and did not depend on the condition, which indicates greater variability of morphological features of SW patterns in this patient. The majority of the SW patterns in P2 had the Sim index below 0.4. The application of this index as an additional feature for classification allowed to eliminate the outliers and improve the performance in P2 even more (S1 = 0.94, S2 = 0.93, S3 = 1.00), while deterioration of the classification performance was observed in P1 in both conditions (before ctDCS: S1 = 0.38, S2 = 0.38 and S3 = 0.99; after ctDCS: S1 = 0.56, S2 = 0.56 and S3 = 1.00).

### 2.5. Impact of Single Features on Classification Performance

**Figure 5:**
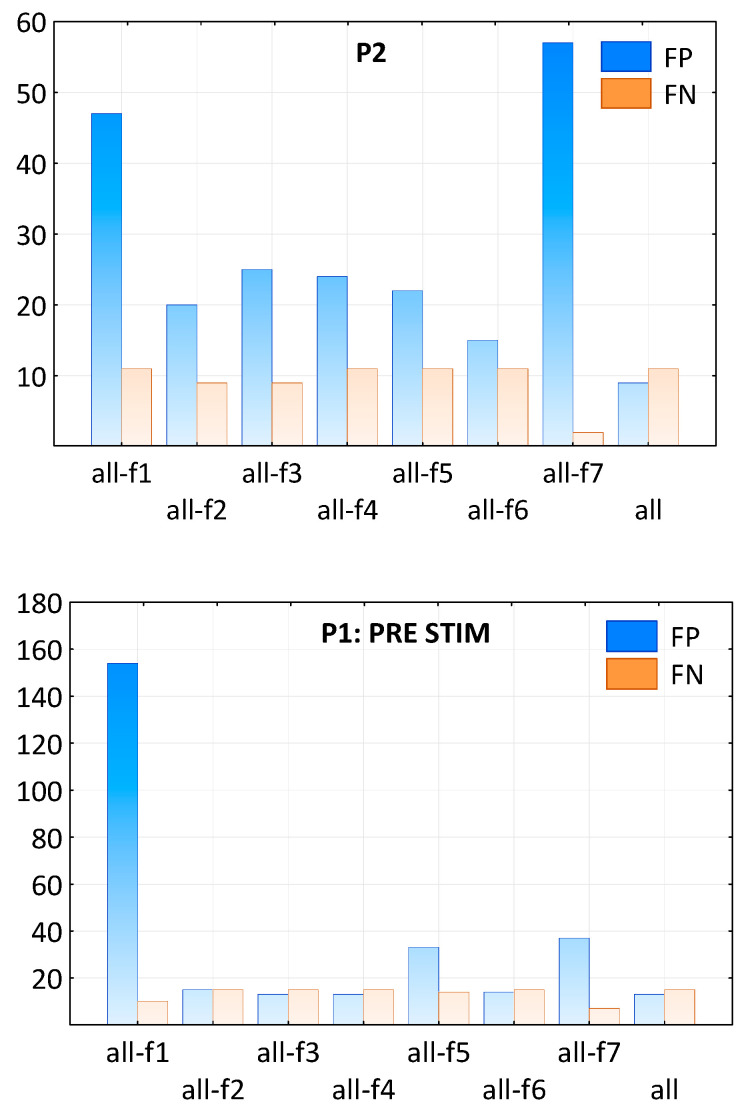

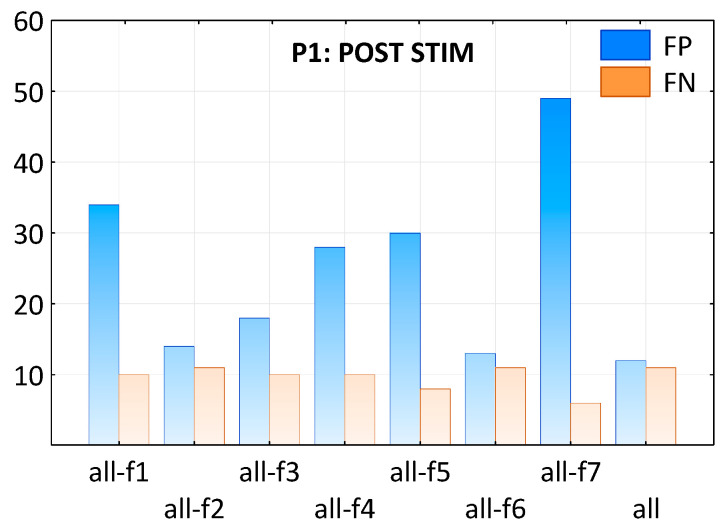
The number of FP and FN patterns as a result of classification with all features compared to the classification with a set without one of the features (f1–f7) in both patients: P1 in two conditions: PRE and POST stDCS, and P2.

The importance of individual features in classification was tested by removing one of them from the whole set for the performance calculation. The number of FP and FN patterns resulting from classification with all features and the classification with a set without one of the features (f1–f7) in both patients and conditions is shown in Figure 5.

**Figure 6:**
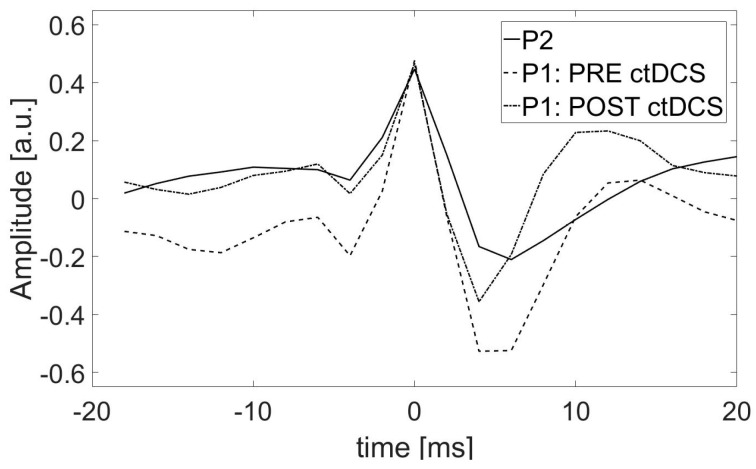
The average SW patterns in patient P1 in two conditions: PRE and POST stDCS and in patient P2. The windows in the range from −20 ms to 20 ms from the spike maximum were considered for averaging all patterns identified in every patient separately.

Interestingly, all features were important for the classification in P1 after ctDCS and in P2, but not in P1 before stimulation. In the latter case, the most important feature was definitely the spike amplitude in the maximum (f1). Moreover, the other two features, the ratio between the rising and falling part of the spike (f5) and the duration of the rising part of the slow wave (f7), were significant in the improvement of the classification result. In contrast to P1 after ctDCS, in both of the other cases (P1 before ctDCS and P2), the most important feature was f7, followed by f1. The shape of SW patterns in P1 after simulation is more similar to that in P2 (see Figure 6), and thus, it can be described by similar features (compare P2 and P1 POST STIM in Figure 5).

### 2.6. Impact of the ctDCS on the Morphology of SW Patterns

A decrease in the number of SW patterns after stimulation was observed (see Table 1, the numbers of TP and FN for P1 in both conditions, PRE STIM and POST STIM). The total number of SW patterns identified by the expert, which is the sum of TP and FN, was equal to 154 and 110 (the first row in Table 1), before and after stimulation, respectively. Moreover, the stimulation caused a change in most of the SW patterns’ features. The significant differences between SW patterns were found for features f2–f6 but not for f1 and f7. The average values of all features and the results of their comparison in P1 for both conditions are shown in Table 2. The values of f2–f5 decreased, while f6 increased after ctDCS. The *p*-values obtained from the Kolmogorov–Smirnov test were reported.

## 3. Discussion

In this study, we proposed a fast and simple method of automatic identification of SW patterns in epileptic EEG based on a few criteria: (1) the simultaneous appearance of spikes on at least two EEG channels; (2) the selection of seven morphological features with values in the ranges determined for SW patterns indicated by an expert; and (3) an appropriate normalization strategy.

Compared to other modern methods of detecting epileptic discharges, allowing for discrimination between healthy, interictal and ictal states [25], our method allows for the detection of single SW patterns and analysis of changes in the morphological structure of these patterns, which leads to a better explanation of the generation of these patterns and their disintegration under the influence of stimulation. Studying the frequency of these patterns in the interictal period may also allow for the prediction of an epileptic seizure.

The algorithm is a modification of our method previously developed to detect similar patterns in volatile anesthesia [21], but the new method of identification of SW patterns in epilepsy is more efficient. Other methods used so far [4,5,6,7,8,9,10,11,12,13,14,22,23,24] did not consider the condition for synchronization of patterns on at least two EEG channels. This criterion is a crucial feature allowing for the efficient identification of SW patterns. To ensure that this condition was met, it was necessary to properly preprocess the EEG signal by (1) resampling to 100 Hz, (2) standardization and (3) min–max normalization. However, the choice of an appropriate normalization strategy is crucial in achieving high accuracy in SW detection. Moreover, our method considered some additional features compared to other algorithms, such as Hjorth’s mobility. Thus, two features not previously considered by other authors, the synchronicity of EEG patterns occurrence on several channels and their characteristic mobility, turned out to be particularly useful for identifying SW patterns.

The larger distribution of the similarity index in P1 may explain the significant deterioration in classification performance due to standardization. This may be due to the fact that standardization can only be applied when the data have a Gaussian distribution, whereas normalization does not have to satisfy this condition. However, normalization is highly influenced by outliers. We have demonstrated that the combination of standardization and min–max normalization gave better results in signals with high pattern similarity. Otherwise, standardization was omitted to improve the classification performance.

Additional factors, such as inter-individual variability of EEG signals and signal recording criteria, like the choice of appropriate montage and the reference electrode, can contribute to the efficiency of classification.

The inter-individual variability has been revealed by the comparison of the similarity indices distributions and the effect of standardization on the classification performance in P1 and P2. The same normalization strategy had a similar effect in P1 independent of condition but not in P2, which is consistent with wider similarity distributions in P1 than in P2. These differences between patients may result from both the individual patient’s EEG fingerprint and the way the signal is recorded. The signal in patient P2 was recorded using the reference electrode at the neck. The placement of the reference electrode in this position, which is distant from the source of epileptic activity, guarantees obtaining a more reliable recording reflecting a true potential distribution of the epileptic activity generator. In contrast, the reference electrode at FCz in patient P1 was placed relatively near the source located at T4, which may have an impact on the higher variability of the SW patterns’ shape.

Nevertheless, the proposed method allowed for the evaluation of the effect of ctDCS therapy. A positive impact of ctDCS therapy was expressed by the decrease in the number of SW patterns after stimulation, which is in line with our previous studies [26] using the same data that showed a reduction in the frequency of seizures after the ctDCS treatment. Furthermore, the beneficial effect of ctDCS therapy was revealed by the decrease in the spike amplitude (4.19 ± 1.08 vs. 4.63 ± 1.02, *p* < 0.010) and the duration of falling part of spike (0.80 ± 0.19 vs. 1.06 ± 0.24, *p* < 0.001; 0.92 ± 0.19 vs. 1.14 ± 0.23, *p* < 0.001), and as a consequence, the decrease in the ratio between rising and falling part of spike (0.59 ± 0.13 vs. 0.66 ± 0.13, *p* < 0.001) and the increase in its mobility representing the mean frequency (1.33 ± 0.23 vs. 1.24 ± 0.21, *p* < 0.040). These results indicate that stimulation causes SW patterns to become less sharp, which is manifested by their lower amplitude and duration. Also, a higher average frequency of patterns occurring in the EEG is characteristic of healthy brain activity. The similarity in the shape of the average SW pattern in P1 after ctDCS therapy and that in P2 may suggest that their health condition is similar. Nevertheless, the patterns’ variability in P1 remained, such as before the therapy.

The proposed method allows for the effective detection of SW patterns and, consequently, the determination of changes in the frequency of patterns occurrence and changes in their morphological features, which allows for the assessment of ctDCS therapy effectiveness and for its personalization.

## 4. Materials and Methods

### 4.1. Patient Recruitment

Two males (20 and 18 years old) participated in this study. For the first patient (P1), the interictal activity was located in the temporal lobe at electrode T4, while for the second patient (P2) at the electrodes O1 and T5. The study was approved by the Ethics Committee of the Campus Bio-Medico University, Rome, Italy (P1) and by the Ethics Committee of the Medical Center for Postgraduate Education, Warsaw, Poland (P2). All patients signed a written informed consent.

### 4.2. ctDCS Treatment

A twenty-year-old drug-resistant patient (P1) underwent ctDCS using a battery-driven stimulator (Schneider Electronic, Gleichen, Germany). A current of 1 mA was delivered for 20 min to two saline-soaked sponge electrodes (5 cm by 7 cm), a cathode was placed over the epileptic focus, and an anode was placed on the opposite side.

### 4.3. EEG Recording

For patient P1, a 15 min EEG was recorded with a sampling frequency of 1000 Hz, and the reference electrode was at FCz before and after ctDCS during the resting state with eyes closed. For patient P2, a 10 min EEG was recorded using a standard 10–20 system with a sampling frequency of 500 Hz and the reference electrode at the neck (nck). The segments with artifacts were rejected manually.

### 4.4. Automatic Detection of SW Patterns

In this study, we developed a new detection method based on morphological features of SW patterns observed in epileptic EEG, which is a modification of our previous algorithm applied for automatic detection of sharp wave–slow wave patterns observed in the EEG data of healthy persons undergoing volatile anesthesia [21].

#### 4.4.1. EEG Preprocessing

Firstly, the EEG signal was preprocessed by (1) resampling to 100 Hz, (2) standardization (c.f. Equation (1)) and (3) min–max normalization in the range [−1, 1] (c.f. Equation (2)).

(1) $$x_{standarized}=\frac{x-mean(x)}{std(x)}$$

(2) $$x_{min-max}=\frac{x-min(x)}{max(x)}-1$$

The resampling to 100 Hz is required for the next step, i.e., the identification of potential SW patterns, to guarantee a correct synchronization between every pair of EEG signals. The min–max normalization allows for eliminating interindividual differences in signal amplitude. Moreover, the impact of standardization on classification performance was studied.

#### 4.4.2. Identification of Potential SW Patterns

Next, the potential SW patterns were identified, considering their synchronization on at least two EEG channels. For this purpose, the 19-channel EEG signal was transformed into 19 sequences of symbols: 1, −1 and 0, corresponding to increase, decrease or lack of amplitude change with an accuracy of 1 μV. The product of every pair of sequences was defined according to the following rules: A*A = A, where A = {1,−1}, while the product of any other pair of symbols is zero. In such a way, a synchronous increase or decrease in the two signals was codified as symbols 1 or −1, respectively. The difference between successive values of such product is equal to 2 or −2 for the synchronously occurring maxima or minima, respectively. Additionally, for better smoothing of the product, the sequences (101), (−10−1), (10−1), (−101) were replaced by sequences (111), (−1−1−1), (1−1−1), (−111), respectively.

**Figure 7:**
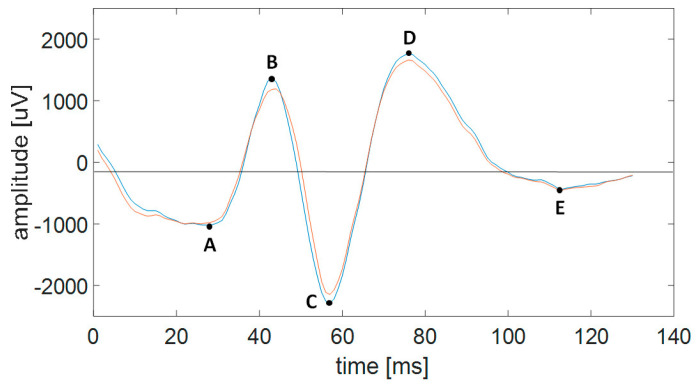
Example of SW pattern on two EEG channels. The spike is from A to C, while the slower wave is from C to E.

Then, the vector of non-zero differences between successive values of the product was created. The potential spikes were identified as one of the following sequences: (−22), (−21), (−12) or (−11) were found in this vector. In such a way, the positions of the successive five minimums and maximums in the SW pattern (points A–E in Figure 7) and the signal amplitudes at these positions were determined.

#### 4.4.3. Features of SW Patterns

We considered only patterns that satisfied the following criteria for features f1–f7: (1) the spike amplitude in the maximum was not smaller than 0.3 a.u. (f1); (2) spike duration, i.e., the interval between points A and C in Figure 7 was from 30 to 70 ms (f2); (3) the amplitude between the spike maximum (points B in Figure 7) and the successive minimum (point C in Figure 7) for at least one from two synchronized EEG channels was not smaller than 0.6 a.u. (f4), and for the second channel was not smaller than 0.5 a.u. (f3); (4) the ratio between the rising and falling part of spike amplitude (the intervals AB and BC in Figure 7) was between 0.35 and 0.9 (f5); (5) the Hjorth’s mobility was not smaller than 0.8 (f6); (6) the rising part of slow wave (the interval CD in Figure 7) lasts more than 50 ms (f7).

The Hjorth’s mobility (M) is defined as:

(3) $$M=\sqrt{\frac{\mathrm{var}(dx/dt)}{\mathrm{var}(x)}}$$

where var(*x*) is the variance of *x*; *dx/dt* is the first derivative of the signal *x* (here, the difference of signal values between successive samples). This feature represents the mean frequency.

#### 4.4.4. Selection of SW Patterns

Next, the potential spikes were sorted in relation to their positions and amplitudes for both synchronized EEG channels. Among the pairs of spikes that appeared at the same time, only those with the maximal amplitudes were taken for further analysis.

### 4.5. Similarity Index

We have introduced a similarity index (Sim) to evaluate the variability of morphological features of the SW patterns, which is characteristic of an individual patient.

The similarity index is defined as the sum of squared errors calculated between a single SW pattern (SW_i_) and the template, i.e., the average SW pattern in the same patient (c.f. Equation (4)). The windows in the range from −20 ms to 20 ms from the spike maximum are considered.

(4) $$Sim(SW_{i})=\sum_{k=t-20}^{t+20}{\left(SW_{i}(k)-mean(SW(k))\right)}^{2}$$

In Figure 6, the average SW patterns are shown for each patient and condition.

## 5. Conclusions

The novelty of the proposed method refers to the application of a combination of a few criteria, such as (1) the simultaneous appearance of spikes on at least two EEG channels; (2) the selection of seven morphological features with values in the ranges determined for SW patterns indicated by an expert; and (3) an appropriate normalization strategy, which allowed for identification of SW patterns in epileptic EEG. The classification performance of the new method for the identification of SW patterns in epilepsy is satisfactory, and thus, it can be useful in the clinical evaluation of the ctDCS therapy effect.

The limitation of this study is the small number of patients and methodological differences in the recording of EEG signals, in particular, the choice of electrode placement and the sampling frequency. Interindividual differences should be taken into consideration as well.

Future studies should include a larger number of patients in a homogeneous group. The dependence on the reference electrode should be investigated. For example, in the case of a discharge focus in the frontal lobe, the use of a frontal reference electrode is particularly unfavorable in the evaluation of SW patterns.

Furthermore, the personalized dependence of the stimulation effect on the number of sessions at longer time intervals should be determined.

## Figures and Tables

**Table 1:** The performance of SW patterns classification. The definition of the numbers of patterns (TP, FP, TN and FN), sensitivity, specificity and selectivity were provided in Appendix A. The results were provided for two patients (P1 and P2) with and without the application of the similarity index. Additionally, a comparison of the results with and without standardization was provided in patient P1. For patient P1, the EEG recording was evaluated in two conditions: before and after ctDCS. The options with the best performance are marked in gray.

Subject	Condition	Similarity Index	Standardization	TP	FP	TN	FN	Sensitivity	Selectivity	Specificity
P1	PRE STIM POST STIM	NO NO	NO NO	139 99	13 12	16033 63778	15 11	0.91 0.89	0.90 0.90	1.00 1.00
	PRE STIM POST STIM	YES YES	NO NO	58 62	94 49	15952 63741	96 48	0.38 0.56	0.38 0.56	0.99 1.00
	PRE STIM POST STIM	NO NO	YES YES	145 104	62 41	15984 63749	9 6	0.70 0.72	0.94 0.95	1.00 1.00
	PRE STIM POST STIM	YES YES	YES YES	114 110	93 35	15953 63755	40 0	0.55 0.76	0.74 1.00	0.99 1.00
P2	-	NO	YES	112	9	29868	11	0.93	0.91	1.00
		YES	YES	114	7	29870	9	0.94	0.93	1.00

**Table 2:** The average values of features in P1 for two conditions (before and after stimulation) and the results of their comparison. The statistically significant values are marked in bold.

Feature	PRE_STIM	POST_STIM	*p*-Value
f1	0.48±0.15	0.47±0.11	*p* < 0.100
**f2**	**4.63 ± 1.02**	**4.19 ± 1.08**	***p* < 0.010**
**f3**	**1.06 ± 0.24**	**0.80 ± 0.19**	***p* < 0.001**
**f4**	**1.14 ± 0.23**	**0.92 ± 0.19**	***p* < 0.001**
**f5**	**0.66 ± 0.13**	**0.59 ± 0.13**	***p* < 0.001**
**f6**	**1.24 ± 0.21**	**1.33 ± 0.23**	***p* < 0.040**
f7	7.60 ± 1.45	7.20 ± 1.27	*p* < 0.100

## Data Availability

Data access may be requested from Aleksander Sobieszek, email: so.aleks.warsaw@gmail.com, and Giovanni Assenza, email: g.assenza@policlinicocampus.it.

## Appendix A

Sensitivity (S1), specificity (S2) and selectivity (S3) are defined as follows:

(A1) $$S1=\frac{TP}{TP+FN}$$

(A2) $$S2=\frac{TN}{TN+FP}$$

(A3) $$S3=\frac{TP}{TP+FP}$$

where TP (true positive) is the number of patterns recognized by the classification system in agreement with the expert evaluation; TN (true negative) is the number of patterns neither recognized by the classification system (i.e., the synchronized patterns that did not satisfy the criteria for features f1–f7), nor by the expert; FP (false positive) is the number of patterns recognized by the classification system but not recognized by the expert; FN (false negative) is the number of patterns recognized by the expert but not recognized by the classification system.
